# Efficacy of Collimated Light Therapy for Seasonal Affective Disorder: study protocol

**DOI:** 10.1192/j.eurpsy.2025.1324

**Published:** 2025-08-26

**Authors:** M. Giordano, G. Longo, L. Orsolini, U. Volpe

**Affiliations:** 1 Unit of Clinical Psychiatry, Department of Clinical Neurosciences/DIMSC, Polytechnic University of Marche, Ancona, Italy

## Abstract

**Introduction:**

Light therapy is a treatment that involves daily exposure to bright light. It is most commonly used to treat seasonal affective disorder (SAD). The standard light therapy regimen for SAD typically involves sitting in front of a light box that emits 10,000 lux of light at a distance of 20 cm from the eyes for 30 minutes per day, preferably in the morning. Sandkühler et al. suggested that increasing the illuminance in light therapy, using a Bright, whole-ROom, All-Day (BROAD) approach, may enhance its effectiveness. Both the standard light therapy regimen and BROAD light therapy involve radiant light sources, which emit light that disperses quickly and produces shadows that vary in size depending on the distance of the object from the light source. This makes them visibly different from sunlight. This study aims to investigate the efficacy of collimated light therapy, which uses a reflective parabola placed behind an LED to generate a parallel light beam, brightly illuminating a whole room in the patient’s home (Collimated Light Therapy), similar to natural sunlight.

**Objectives:**

H1.0) To measure the difference in symptom severity between baseline and after 4 weeks of treatment using the SIGH-SAD questionnaire on SAD symptoms. H1.1) To assess the difference in symptom severity between baseline and after 4 weeks of treatment using the BDI-SAD. H1.2) To determine the fraction of patients in remission after 4 weeks.

**Methods:**

This study includes adult patients diagnosed with SAD according to DSM-5-TR criteria. Participants must be at home for at least 6 hours in the morning and afternoon (before 19:00) on at least 5 days per week. Exclusion criteria include a history of manic episodes, light therapy in the previous 4 months, and recent changes in antidepressant medication. We will test hypotheses H1.0 and H1.1 using independent-sample t-tests to compare average scores on the SIGH-SAD and BDI-SAD scales between the collimated light therapy and standard light therapy groups after four weeks. Hypothesis H1.2 will be tested with a chi-square test for association, comparing remission rates between the two groups after two and four weeks.

**Results:**

Our hypotheses are: (1) Home-based collimated light therapy for at least 6 hours/day reduces winter depression symptoms more than standard light therapy (light box, 10,000 lux, 30 minutes/day) after four weeks, as measured by the SIGH-SAD. (2) After four weeks, collimated light therapy shows greater improvement in symptoms as measured by the BDI-SAD. (3) Collimated light therapy results in a higher fraction of patients in remission after four weeks.

**Conclusions:**

This trial is the first RCT to compare the efficacy of collimated light therapy with standard light therapy for treating SAD. This groundbreaking trial could open a new therapeutic frontier, offering a potentially more effective option for patients suffering from SAD.

**Disclosure of Interest:**

None Declared

